# Association Between Body Mass Index and All-Cause Mortality in a Prospective Cohort of Southern Chinese Adults Without Morbid Obesity

**DOI:** 10.3389/fphys.2022.857787

**Published:** 2022-04-25

**Authors:** Feng Hu, Jianduan Cheng, Yun Yu, Tao Wang, Wei Zhou, Chao Yu, Lingjuan Zhu, Huihui Bao, Xiaoshu Cheng

**Affiliations:** ^1^ Department of Cardiovascular Medicine, the Second Affiliated Hospital of Nanchang University, Nanchang, China; ^2^ Jiangxi Provincial Cardiovascular Disease Clinical Medical Research Center, Nanchang, China; ^3^ Jiangxi Sub-Center of National Clinical Research Center for Cardiovascular Diseases, Nanchang, China; ^4^ Wuyuan Hospital of Traditional Chinese Medicine, Wuyuan, China; ^5^ Center for Prevention and Treatment of Cardiovascular Diseases, the Second Affiliated Hospital of Nanchang University, Nanchang, China

**Keywords:** all-cause mortality, body mass index, Chinese, obesity paradox, adults

## Abstract

**Objective:** This prospective study examined the relationship between body mass index (BMI) and all-cause mortality in Chinese adults without morbid obesity.

**Methods:** We prospectively examined the relationship between BMI and all-cause mortality in 12,608 Southern Chinese adults with age ≥35 years who participated in the National Key R&D Program from 2013–2014 to 2019–2020. Cox proportional hazards models were used to examine the association between BMI and all-cause mortality.

**Results:** The prevalence of being underweight, normal weight, overweight and having moderate obesity was 7.36%, 55.83%, 28.51% and 8.31%, respectively. A total of 683 (5.65%) deaths occurred during a median follow-up period of 5.61 years. The Cox proportional hazards models indicated that a continuous BMI level was negatively associated with all-cause mortality [adjusted-hazard ratio (HR) per 1 kg/m^2^ increase: 0.96, 95% *CI* 0.93 to 0.98, *p* < 0.001]. Furthermore, the HRs of all-cause mortality in the underweight, overweight and moderate obesity groups were 1.31 (1.05, 1.64), 0.89 (0.73, 1.08) and 0.64 (0.44, 0.92), respectively in the confounder model relative to the normal weight group. Survival analysis further confirmed this inverse association of the four BMI categories with mortality.

**Conclusion:** BMI was negatively associated with all-cause mortality in southern Chinese adults without morbid obesity. Compared to the normal weight category, adults in the moderate obesity category had lower all-cause mortality, whereas being underweight was associated with increased all-cause mortality.

## Introduction

Obesity, which is a global public health concern, increases the incidence of various chronic diseases (e.g., cardiovascular disease and cancer) and mortality risk (Berrington de Gonzalez et al., 2010; Di Angelantonio et al., 2016; Blüher, 2019). Body mass index (BMI), which is defined as weight in kilograms divided by height in meters squared, is used to identify obesity. For Chinese adults, a BMI of 24.0–27.9 kg/m^2^ is defined as overweight and a BMI of 28.0 kg/m^2^ or higher is defined as obese (Zhou, 2002). Furthermore, obesity is further categorized into moderately obese (BMI of 28.0–34.9 kg/m^2^) and severe (morbidly) obese (BMI of ≥ 35.0 kg/m^2^), the latter accompanied with a higher all-cause mortality rate (Chiang et al., 2019).

Although large-scale, long-term studies have consistently demonstrated an increased risk of all-cause mortality in morbidly obese individuals (Berrington de Gonzalez et al., 2010; Di Angelantonio et al., 2016; Khan et al., 2018), the relationship between overweight-moderate obesity and the risk of mortality is controversial (Sasazuki et al., 2011; Chen et al., 2012; Flegal et al., 2013; Oakkar et al., 2015; Srikanthan et al., 2016; Sun et al., 2016; Wang et al., 2016; Chen et al., 2017; Kwon et al., 2017; Ladhani et al., 2017; Lamelas et al., 2017; Ball et al., 2018; Dhalwani et al., 2018; Gajalakshmi et al., 2018; Jayedi and Shab-Bidar, 2018; Kim et al., 2018; Lee et al., 2018; Lv et al., 2018; Park et al., 2018; Wang et al., 2018; Xia et al., 2019). Existing evidence suggests that in comparison with being normal weight, such conditions are potentially not associated with higher mortality and are even possibly associated with lower all-cause mortality in the general population (Sasazuki et al., 2011; Chen et al., 2012; Flegal et al., 2013; Oakkar et al., 2015; Sun et al., 2016; Wang et al., 2016; Chen et al., 2017; Dhalwani et al., 2018; Gajalakshmi et al., 2018; Kim et al., 2018; Lee et al., 2018; Lv et al., 2018; Park et al., 2018; Wang et al., 2018), those with diabetes mellitus (Kwon et al., 2017; Dhalwani et al., 2018), chronic kidney disease (Ladhani et al., 2017), and cardiovascular diseases (CVDs) (Srikanthan et al., 2016; Xia et al., 2019) including heart failure with preserved or reduced ejection fraction (HFpEF or HFrEF) (Xia et al., 2019), hypertension (Jayedi and Shab-Bidar, 2018), atrial fibrillation (Ball et al., 2018) and coronary artery disease (Lamelas et al., 2017; Xia et al., 2019). Between 2002 and 2012, prevalence of overweight and obesity have increased rapidly in the past 4 decades, and the latest national prevalence estimates for 2015-19, based on Chinese criteria, were 34.3% for overweight and 16.4% for obesity in adults (Pan et al., 2021). Therefore, we prospectively examined the relationship between BMI and all-cause mortality in Chinese adults without morbid obesity.

## Methods

### Study Design and Population

**FIGURE 1:**
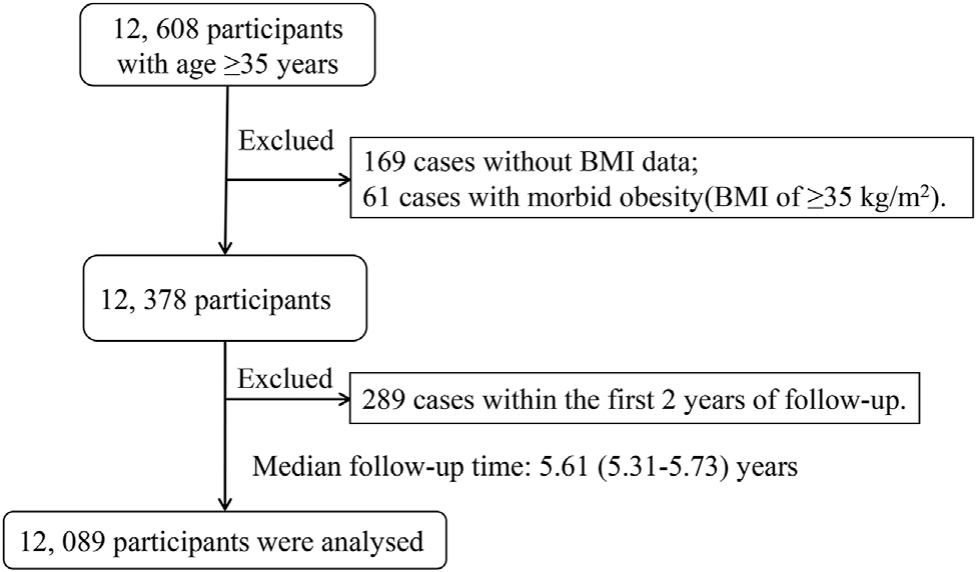
Flow chart of this analysis. Abbreviations: BMI, body mass index.

This cohort study was supported by the National Key R&D Program in the Twelfth Five-year Plan (No. 2011BAI11B01). Detailed information regarding the background, purpose, methodologies, and design of the research has been described in detail in previous publications (Wang et al., 2014; Hu et al., 2017a; Hu et al., 2017b; Hu et al., 2018a; Hu et al., 2018b). The 15,269 participants completed the baseline investigation between November 1, 2013 and August 31, 2014 (Wang et al., 2014). We followed 12,608 participants with age ≥35 years at baseline between July 1, 2019 and October 1, 2020. The 12,608 participants were followed up by means of telephone follow-up, death certificate diagnoses from Jiangxi Provincial Center for Disease Control and Prevention (responsible for monitoring provincial causes of death), and a follow-up visit with local public health doctor or village doctor. There were no lost to follow-up among all participants. The median follow-up duration was 5.61 (5.31–5.73) years. After excluding 169 cases without BMI data, 61 cases with morbid obesity (BMI of ≥ 35 kg/m^2^) and 289 cases within the first 2 years of follow-up, a final total of 12,089 participants were included in the analysis (Figure 1).

### Data Collection Procedure

Participants completed a questionnaire conducted through face-to-face talks by trained staff. Information acquired by the questionnaire included general data (such as age, sex and district), lifestyles (such as smoking and alcohol habits, as well as physical activity levels), medication usage, and medical history (such as hypertension, myocardial infarction and stroke). Current smokers were defined as having smoked at least one cigarette per day for 6 months or more (Hu et al., 2017b). Current drinkers were defined as drinking alcohol at least one time per week during the previous year (Hu et al., 2017b).

The anthropometric measurements included systolic blood pressure (SBP), diastolic blood pressure (DBP), height, and body weight. BP was measured using the Omron HBP-1300 Professional Portable Blood Pressure Monitor (Kyoto, Japan) three times on the right arm supported at the heart level after the participants were allowed to rest for 5 min, with a 30-s interval between measurements. SBP or DBP was defined as the average of the three SBP or DBP readings. Hypertension was defined as SBP ≥140 mmHg and/or DBP ≥90 mmHg or currently receiving or having received treatment for hypertension (Wang et al., 2014; Hu et al., 2017a). Waist circumference was measured (to the nearest 0.5 cm) by placing the measuring tape at the midpoint between the lower margin of the last rib and the top of the hip bone (at the level of umbilicus) at the end of expiration (Hu et al., 2017a). Individuals were in an upright position with the abdomen relaxed at the end of gentle expiration. Body weight without heavy clothing was measured using a weight measurement device (V- BODY HBF-371, Omron, Kyoto, Japan) (Hu et al., 2017a). Height was measured without shoes using a standard right-angle device and a fixed measurement tape (to the nearest 0.5 cm) (Hu et al., 2017a). BMI was calculated as the weight in kilograms divided by height in meters squared (kg/m^2^) (Hu et al., 2017a). Based on BMI (kg/m^2^), participants were categorized as underweight (< 18.5), normal (18.5–23.9), overweight (24–27.9) and obese (≥ 28) (Zhou, 2002). Furthermore, obesity is further categorized into moderate obesity (BMI of 28–34.9 kg/m^2^) and severe morbid obesity (BMI of ≥35 kg/m^2^) (Chiang et al., 2019). In this analysis, normal BMI was used as reference group.

### Mortality Follow-Up

Survival status was ascertained during the follow-up survey between July 1, 2019 and October 1, 2020, assessing whether subjects died and the date of death, completed the study or were lost to follow-up. Cause of death was ascertained by means of telephone follow-up, death certificate diagnoses from Jiangxi Provincial Center for Disease Control and Prevention, and a follow-up visit with local public health doctor or village doctor. The cause of death were further categorized to “stroke, heart disease, malignant tumor, respiratory failure, others and unknown.”

### Statistical Analysis

Continuous variables are presented as the mean ± standard deviation (SD) and are compared using the one-way analysis of variance or the Mann–Whitney *U* test, depending on whether the quantitative data were consistent with a normal distribution. Categorical variables were expressed as count (percentage), differences between groups were measured by chi-square test.

**FIGURE 2:**
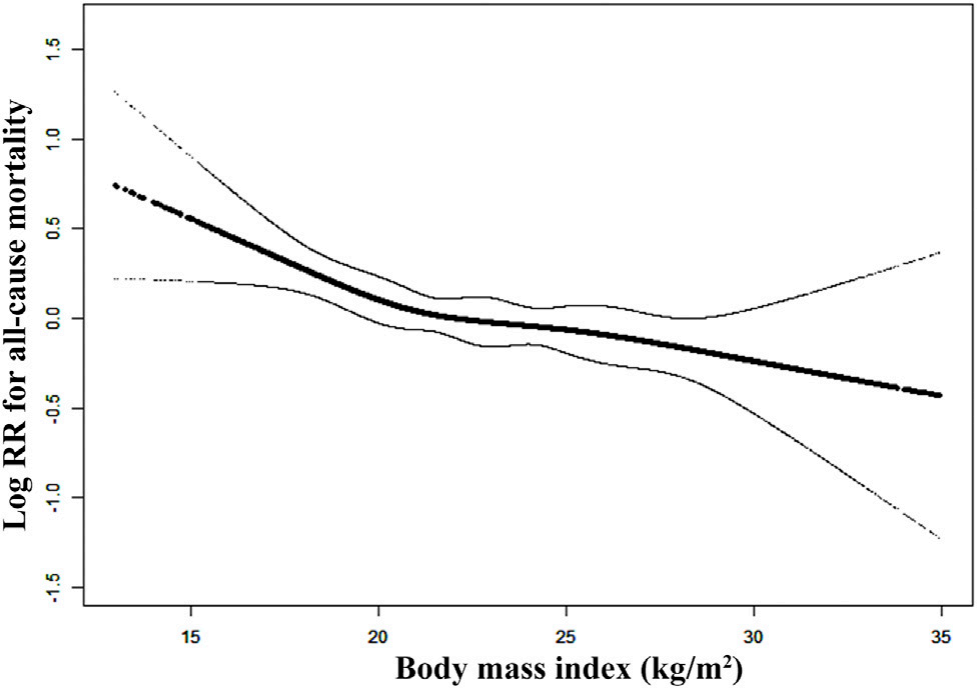
Smooth curve of correlation between body mass index level and all-cause mortality. Note: Smooth curve adjusted for age, sex, current smokers and drinkers, SBP and DBP.

Secondly, to address the linearity or not between the continuous BMI level and the risk of death, a Cox proportional hazards ratio (HR) model with cubic spline functions and smooth curve fitting (restricted cubic spline method) were performed (Figure 2). This ordinate was performed by logarithmic transformed data, where log (relative risk, RR) can be transformed to a relative risk by taking antilog. Then, we used three different Cox proportional hazards models to examine the associations between BMI and all-cause or cardiovascular mortality. The crude model was not adjusted for any confounder. The model Ⅰ was adjusted for age, sex. The model Ⅱ was confounder model, which selected covariates including age, sex, current smokers and drinkers, SBP and DBP. We considered the confounder model to be the main model (Farrar et al., 2015). Linear trend tests were realized by entering the median value of each category of BMI as a continuous variable. Furthermore, the effect of the quartiles of BMI level on death events was also assessed by Kaplan-Meier curves (Xu et al., 2017). In addition, subgroup analysis was executed by stratified and interaction test to investigate the robustness between quartiles of BMI and all-cause mortality.

The R package (http://www.R-project.org, version 3.4.3) and the Empower (www.empowerstats.com) carried out these statistical analyses. A two-tailed *p* < 0.05 was considered to be statistically significant.

## Results

### Patient Characteristics at Baseline

This analysis included 12,089 Chinese adults (age: 58.84 ± 13.12 years, range 35–97 years; males, 40.36%), and the prevalence of underweight, normal weight, overweight and moderate obesity was 7.36%, 55.83%, 28.51% and 8.31%, respectively. The clinical baseline characteristics of the study participants are presented in Table 1 according to the different BMI categories. There were significant differences in age, SBP, DBP, weight, waist circumference, percentage of urban residents, physical activity levels, current smokers, prevalence of hypertension and antihypertensive medications among the four categories of different BMI level.

**TABLE 1:** Baseline characteristics of the study population according to different BMI categories.

Characteristics	Total subjects	Classification of BMI				*p*-value
		Underweight	Normal weight	Overweight	Moderate obesity	
Number of subjects (n)	12089	890	6749	3446	1004	<0.001
Age (years)	58.84 ± 13.21	65.69 ± 14.15	59.03 ± 13.50	57.22 ± 12.01	57.03 ± 12.26	
Male, n (%)	4879 (40.36%)	390 (43.82%)	2706 (40.09%)	1364 (39.58%)	419 (41.73%)	0.099
SBP^a^ (mmHg)	127.86 ± 19.46	124.83 ± 20.85	125.77 ± 19.18	130.76 ± 18.95	134.78 ± 18.84	<0.001
DBP^a^ (mmHg)	74.78 ± 10.71	71.36 ± 9.88	73.44 ± 10.25	76.90 ± 10.82	79.58 ± 11.22	<0.001
Height (m)	1.56 ± 0.08	1.56 ± 0.09	1.56 ± 0.08	1.56 ± 0.09	1.56 ± 0.09	0.146
Weight (kg)	56.45 ± 10.42	42.10 ± 5.36	52.56 ± 6.73	62.89 ± 7.50	73.20 ± 8.86	<0.001
BMI (kg/m2)	23.07 ± 3.37	17.31 ± 1.03	21.50 ± 1.48	25.65 ± 1.12	29.83 ± 1.59	<0.001
Waist circumference (cm)	79.97 ± 9.28	69.83 ± 7.35	76.44 ± 6.55	85.59 ± 6.81	93.48 ± 8.69	<0.001
Urban residents, n (%)	6191 (51.21%)	419 (47.08%)	3274 (48.51%)	1910 (55.43%)	588 (58.57%)	<0.001
Physical activity levels^a^, n (%)						<0.001
Low	1836 (15.23%)	165 (18.64%)	1016 (15.09%)	469 (13.64%)	186 (18.58%)	
Middle	3201 (26.55%)	262 (29.60%)	1775 (26.36%)	875 (25.45%)	289 (28.87%)	
High	7021 (58.23%)	458 (51.75%)	3943 (58.55%)	2094 (60.91%)	526 (52.55%)	
Current smokers^a^, n (%)	2376 (19.70%)	206 (23.17%)	1369 (20.35%)	619 (17.99%)	182 (18.18%)	<0.001
Current drinkers^a^, n (%)	3020 (25.07%)	193 (21.78%)	1680 (24.98%)	892 (25.97%)	255 (25.53%)	0.082
Hypertension, n (%)	4114 (34.03%)	254 (28.54%)	1988 (29.46%)	1380 (40.05%)	492 (49.00%)	<0.001
History of myocardial infarction, n (%)	85 (0.70%)	7 (0.79%)	47 (0.70%)	22 (0.64%)	9 (0.90%)	0.841
History of stroke, n (%)	205 (1.70%)	10 (1.12%)	108 (1.60%)	69 (2.00%)	18 (1.79%)	0.249
Antihypertensive medications, n (%)	1089 (9.01%)	42 (4.72%)	459 (6.80%)	427 (12.39%)	161 (16.04%)	<0.001
Diuretics	15 (0.12%)	0 (0.00%)	5 (0.07%)	6 (0.17%)	4 (0.40%)	0.026
ACEIs or ARBs, n (%)	297 (2.46%)	16 (1.80%)	132 (1.96%)	108 (3.13%)	41 (4.08%)	<0.001
Beta blockers, n (%)	57 (0.47%)	2 (0.22%)	20 (0.30%)	24 (0.70%)	11 (1.10%)	<0.001
Calcium channel blockers, n (%)	763 (6.31%)	24 (2.70%)	314 (4.65%)	299 (8.68%)	126 (12.55%)	<0.001
Other agents, n (%)	123 (1.02%)	2 (0.22%)	54 (0.80%)	52 (1.51%)	15 (1.49%)	<0.001

Abbreviations: BMI, body mass index; SBP, systolic blood pressure; DBP, diastolic blood pressure; ACEIs, angiotensin-converting enzyme inhibitors; ARBs, angiotensin receptor blockers.

a The number of missing variables: SBP (47); DBP (53); Physical activity levels (Hu et al., 2018b); Current smokers (Hu et al., 2018b); Current drinkers (Tsujimoto and Kajio, 2017).

### Association Between BMI and All-Cause Mortality

A total of 683 (5.65%) deaths occurred during a median follow-up period of 5.61 years. The all-cause mortality varied substantially among the four categories of different BMI level (*p* < 0.001). The numbers of deaths were 102, 400, 149 and 32, with rates of death from all causes at 11.46%, 5.93%, 4.32% and 3.19%, according to the four categories of different BMI level, respectively (Table 2).

**TABLE 2:** All-cause mortality of the study population by different BMI categories.

Characteristics	Total subjects	BMI categories				*p*-value
		Underweight	Normal weight	Overweight	Moderate obesity	
Median follow-up time, years	5.61 (5.31–5.73)	5.62 (5.31–5.73)	5.61 (5.32–5.73)	5.59 (5.30–5.74)	5.59 (5.29–5.74)	0.189
All-cause mortality, n (%)	683 (5.65%)	102 (11.46%)	400 (5.93%)	149 (4.32%)	32 (3.19%)	<0.001
Cause of death, n (%)						<0.001
Stroke	102 (0.84%)	16 (1.80%)	59 (0.87%)	21 (0.61%)	6 (0.60%)	
Heart disease	218 (1.80%)	37 (4.16%)	131 (1.94%)	41 (1.19%)	9 (0.90%)	
Malignant tumor	52 (0.43%)	8 (0.90%)	27 (0.40%)	13 (0.38%)	4 (0.40%)	
Respiratory failure	90 (0.74%)	16 (1.80%)	49 (0.73%)	21 (0.61%)	4 (0.40%)	
Others	112 (0.93%)	8 (0.90%)	69 (1.02%)	28 (0.81%)	7 (0.70%)	
Unknown	109 (0.90%)	17 (1.91%)	65 (0.96%)	25 (0.73%)	2 (0.20%)	

Abbreviations: BMI, body mass index.

**FIGURE 3:**
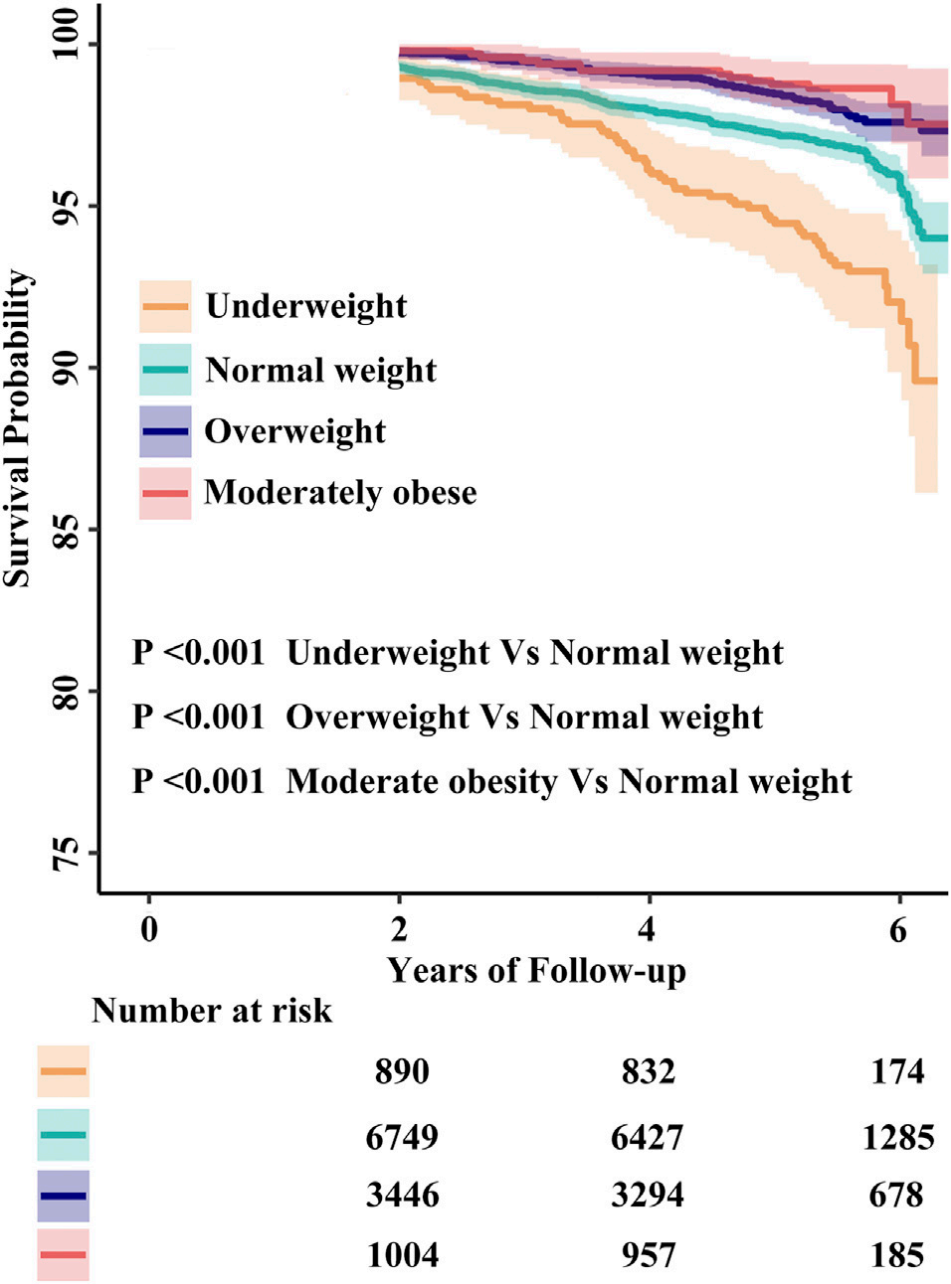
The cumulative hazards of all-cause mortality according to quartiles of body mass index. Abbreviations: BMI, body mass index.

Restricted cubic spline suggested that there was a monotonically decreasing relationship between BMI level and the risk of all-cause death (log-likelihood ratio test <0.001, Figure 2). Cox proportional hazards models also indicated that BMI level was negatively associated with all-cause mortality (adjusted- HR per 1 kg/m^2^ increase in the confounder model: 0.96, 95% CI 0.93 to 0.98, *p* < 0.001, Table 3). The HRs of all-cause mortality in the underweight, overweight and moderate obesity groups were 1.31 (1.05, 1.64), 0.89 (0.73, 1.08) and 0.64 (0.44, 0.92), respectively, in the confounder model, relative to the normal weight group (Table 3). Compared to the normal weight group, the moderate obesity group had lower all-cause mortality, whereas being underweight was associated with significantly increased mortality. Survival analysis further confirmed this negative association of the four BMI categories with all-cause mortality (Kaplan–Meier, log-rank *p* < 0.001, *p* < 0.001, and *p* < 0.001 for the underweight, overweight and moderate obesity groups relative to the normal weight group, respectively; Figure 3).

**TABLE 3:** Hazard ratios of different BMI categories for all-cause mortality.

Variables	Event, n (%)	Crude model		Model Ⅰ		Model Ⅱ	
		HR (95%CI)	*p*-value	HR (95%CI)	*p*-value	HR (95%CI)	*p*-value
BMI							
Per 1 kg/m^2^ increase	683 (5.65%)	0.90 (0.88, 0.92)	<0.001	0.96 (0.94, 0.98)	0.001	0.96 (0.93, 0.98)	<0.001
Classification of BMI							
Underweight	102 (11.46%)	1.98 (1.60, 2.47)	<0.001	1.26 (1.01, 1.57)	0.039	1.31 (1.05, 1.64)	0.015
Normal weight	400 (5.93%)	*Ref*		*Ref*		*Ref*	
Overweight	149 (4.32%)	0.72 (0.60, 0.87)	<0.001	0.91 (0.75, 1.10)	0.336	0.89 (0.73, 1.08)	0.234
Moderate obesity	32 (3.19%)	0.52 (0.36, 0.75)	<0.001	0.66 (0.46, 0.95)	0.025	0.64 (0.44, 0.92)	0.016
P for trend		<0.001		<0.001		<0.001	

Abbreviations: BMI, body mass index; *Ref*, reference; *HR*, hazard ratio; *CI*, confidence interval. Model Ⅰ adjusted for age and sex. Model Ⅱ adjusted for age, sex, current smokers and drinkers, SBP and DBP.

### Subgroup Analyses by Potential Effect Modifiers

**FIGURE 4:**
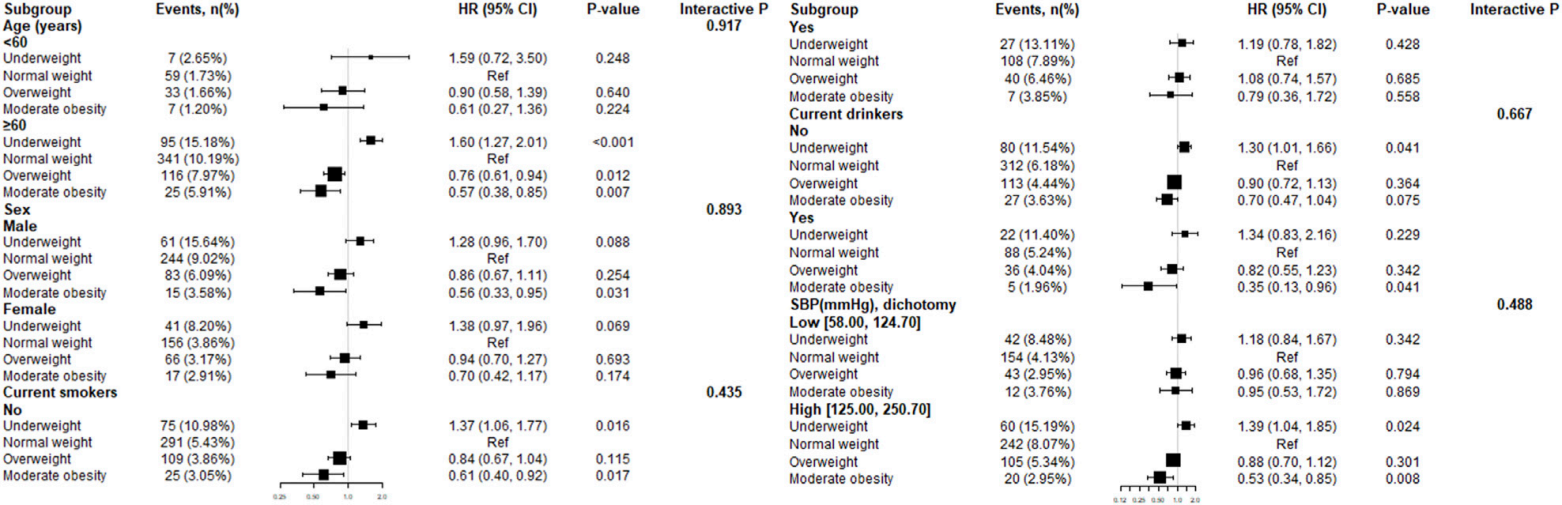
Effect size of the different body mass index categories on all-cause mortality in prespecified and exploratory subgroups. Note: Each stratification adjusted for age, sex, current smokers and drinkers, SBP and DBP except the subgroup variable. Abbreviations: Ref = reference; HR = hazard ratio; CI = confidence interval.

To explore whether this inverse association between BMI and all-cause mortality were still stable among different subgroups, we conducted the stratified and interaction analyses. The subgroup analyses showed that there were not statistically significantly interactions between the different BMI categories and all-cause mortality in any of the subgroups, including age (<60 vs. ≥60 years), sex (male vs. female), current smokers (no vs. yes), current drinkers (no vs. yes) and SBP dichotomy (low vs. high) (Figure 4).

## Discussion

This study presents comprehensive estimates of the association of different BMI categories with all-cause mortality in southern Chinese adults. According to the results presented herein, moderate obesity was associated with significantly lower all-cause mortality relative to being normal weight, whereas being underweight was associated with increased all-cause mortality.

Our findings are highly consistent with previous results of an international multicenter systematic review (Flegal et al., 2013) and a prospective Chinese cohort study (Wang et al., 2018). These results showed a protective role of obese has stirred wide disputes in recent years. Previous studies have found that the relationship of BMI level with overall mortality was irrelevant (Gajalakshmi et al., 2018) or U-shaped (Oakkar et al., 2015) among Chinese. A prospective cohort study found a J-shaped relation between BMI and cause-specific mortality in the elderly population of Taiwan (Lin et al., 2021). There was a U-shape relationship between BMI and all-cause death in patients with serious coronary artery diseases, with increased risks among both underweight and morbid obesity patients (Feng et al., 2021). On the one hand, potential interpretations in terms of this protective effect (Flegal et al., 2013) included prior clinical manifestations and better medical care, as well as enhanced body fat reserves in the obesity group.

On the other hand, there were some factors that should be taken into serious consideration when explaining this counterintuitive result, which included confounding and selection bias (Berrington de Gonzalez et al., 2010; Logue et al., 2011; Robinson et al., 2014; Di Angelantonio et al., 2016; Xia et al., 2019) and the scientific rationality of the use of BMI to define obesity (Gonzalez et al., 2017). For instance, smoking is a common confounding bias (Xia et al., 2019). Compared to overall population, the mortality risk in overweight and obese groups was enhanced among healthy white adults who never smoked (Berrington de Gonzalez et al., 2010). Likewise, dependable evaluations of the causal association between BMI level and mortality should be restricted to participants without pre-existing chronic diseases to reduce confounding bias (Di Angelantonio et al., 2016). Furthermore, obesity group would be unlikely to participate in these cohort studies because of their increased risk of cardiovascular events (Logue et al., 2011; Robinson et al., 2014).

Finally, although BMI was widely applied to identify obesity, it did not present the muscle-fat ratio, or explain the sex gap in the allocation of subcutaneous and visceral fat (Gonzalez et al., 2017). Skeletal muscle plays an important role in glucose metabolism and muscle mass was correlated with improved survival (Srikanthan and Karlamangla, 2014; Chuang et al., 2016; Srikanthan et al., 2016). Moreover, recent data have indicated that overall mortality was remarkably higher in heart failure in the population of people with visceral obesity referred to the standard waistline (Tsujimoto and Kajio, 2017). These results suggested the importance of body composition assessment in the prediction of total mortality (Srikanthan et al., 2016).

Taking into account that potential reverse causation from pre-existing diagnosed or undiagnosed disease, our study excluded subjects within the first 2 years of follow-up. Our study has several limitations. First, BMI cannot reflect body composition (Gonzalez et al., 2017). Secondly, within a relatively short duration of follow-up, fewer deaths restricted the statistical performance to evaluate the relationship of BMI level with all-cause mortality. Compared to the other BMI groups, individuals in the underweight group were older and showed lower physical activity, which might indicate poorer health status at baseline. Finally, baseline information did not contain cancer and more detailed smoking status (never, former, current) as well as smoking variables (cigarettes per day, duration of smoking, and time since smoking cessation), which could lead to confounding bias (Berrington de Gonzalez et al., 2010; Aune et al., 2016; Xia et al., 2019).

In conclusion, BMI was negatively associated with all-cause mortality in southern Chinese adults without morbid obesity. Compared to the normal weight category, adults in the moderate obesity category had lower all-cause mortality, whereas being underweight was associated with increased all-cause mortality.

## Data Availability

The original contributions presented in the study are included in the article/Supplementary Material, further inquiries can be directed to the corresponding authors.
